# The wrong Spirochaete? Acute kidney injury in a returning traveller with syphilis – a case report

**DOI:** 10.1186/s12879-020-05418-4

**Published:** 2020-09-21

**Authors:** Hannah Cook, Mark Gompels

**Affiliations:** Immunology, North Bristol NHS Trust, Southmead Road, Bristol, BS10 5NB UK

**Keywords:** Case report, Acute kidney injury, Syphilis, HIV, Acute tubular necrosis

## Abstract

**Background:**

Syphilis has seen an increased incidence in recent years and can have serious and irreversible consequences if left un-diagnosed and untreated. This case report describes a presentation of syphilis and acute kidney injury – a scenario sparsely described in existing literature.

**Case presentation:**

This 43-year old Man who has Sex with Men (MSM) presented to the emergency department with a 3-week history of vomiting and headaches, progressing to include pyrexia. These symptoms started following his return from a 2-week cruise in Central America throughout which he had been well. He had a background of well-controlled human immunodeficiency virus (HIV).

On admission he had an Acute Kidney Injury (AKI) stage 3, without hydronephrosis, presumed to be pre-renal. Leptospirosis, the main differential, was negative serologically. ‘Pyrexia of unknown origin’ testing was performed, and cefuroxime commenced. Later in the admission, syphilis testing indicated an acute infection and he completed a full treatment course of benzylpenicillin. This, alongside intravenous fluids, resulted in symptom and renal resolution in 9 days and restoration of renal function.

**Conclusions:**

Renal complications in syphilis are rare, furthermore the majority of those documented occur in latent syphilis and are irreversible. There are limited numbers of other documented cases of AKI in acute syphilis, which like the gentleman in this case were reversible and did not lead to permanent kidney damage.

This case adds to the knowledge base of AKI in initial presentation of syphilis. It also demonstrates not only the importance of taking a sexual history in patients with new infective symptoms but that testing for syphilis in at-risk groups regardless of history should be performed given its rising incidence. These considerations by physicians can lead to prompt diagnosis and management of syphilis and improve patient care and long-term outcomes.

## Background

Syphilis is a disease caused by the spirochete treponema pallidum [1] and can be acquired (most commonly sexually; also haematologically transmitted) [2] or congenital (passed in utero) [3]. Its presentation stages are divided into primary, secondary, latent and tertiary [1]. Primary takes the form of a single painless ulcer (chancre) with localised lymphadenopathy [1]; secondary involves multiple systems and can include a non-pruritic, maculo-papular rash on the palms/soles, condylomata lata, fever, malaise, headache and generalised lymphadenopathy [2]. These two stages are the most infective [2]. Latent syphilis is symptomless but serologically diagnosable, whilst tertiary presents as neurological, cardiovascular or gummatous syphilis [2].

Syphilis has re-emerged in recent years, in the UK [4]. In 2018 it saw a 5% total increase in incidence from 2017 to 7541 new cases [4]. A particular resurgence can be seen in younger people, rising in 15–19-year olds by 22% and men who have sex with men (MSMs) by 61% between 2017 and 18 [2]. In recognition of this, Public Health England have published a National Syphilis Action Plan to combat increased rates of transmission [4].

This increasing disease burden, coupled with the serious and irreversible consequences if left undiagnosed and untreated, demonstrate the importance of clinicians being aware of syphilis’ aetiology, management and consideration as a differential diagnosis where appropriate.

This case report investigates a gentleman with a first presentation of syphilis and concurrent acute kidney injury (AKI). Although glomerulonephritis and nephrotic syndrome are mentioned as rarer manifestations of secondary syphilis by the National Institute of Clinical Excellence (NICE) [2], there are few documented case studies of such presentations. Recognising this association is important so that the correct early antibiotic therapy can be administered to prevent lasting kidney impairment and maximise complete recovery. Furthermore, being aware of less typical presentations of syphilis allows diagnosis at earlier stages and prevents progression to latent and tertiary disease.

## Case presentation

A 43-year old MSM presented to hospital emergency department in January 2018 with pyrexia, headaches and vomiting. He was investigated for a travel related fever having returned from a 14-day cruise in Central America 1 week prior to becoming unwell.

In the 3 weeks prior to admission, he developed headaches associated with painful eyes, malaise and myalgia. The headaches increased in severity and after 10 days he began vomiting. He experienced no diarrhoea, cough, rashes or joint pain. He was prescribed naproxen from his General Practitioner to try and alleviate his symptoms but only took 3 tablets. After 1 month of these symptoms, he developed a fever of 39 degrees and rigors. Despite his headaches being more severe, he did not notice any photophobia or neck stiffness. He had no other systemic symptoms, no scleral changes, was neurologically normal and on systems review reported just 1 episode of malodorous urine.

He had received no vaccinations pre-travel and took no malarial prophylaxis. During the trip he was mainly sightseeing, with some outdoor activities such as river swimming. He sustained multiple mosquito bites around the ankles, being bitten from the first day of the holiday. Whilst away, however, he remained well.

He was diagnosed with Human Immunodeficiency Virus (HIV) in 2013, this was well-controlled with a viral load of under 40 and a CD4 count of 940. He never experienced any complications of HIV. His current retroviral therapy was Truvada and Doravirine. He reported only having had sexual contact with his regular male partner, who was well. He had tested negative for syphilis in November 2016.

## Management and outcome

Blood tests revealed a stage 3 AKI with a creatinine of 501 mg/dL, Urea 12.4 mg/dL and glomerular filtration rate (eGFR) 20 ml/min/1.73 m^2^. Given the history of vomiting and fever, he was given intravenous fluids and his anti-retro-virals (ARVs) were withheld due to poor renal function. Ultrasound revealed no hydronephrosis of the kidneys and a urine dip showed glycosuria (HBA1c and oral glucose tolerance tests were negative) and no significant proteinuria. A renal review concluded that the AKI was most likely due to acute tubular necrosis which was only partially responsive to intravenous fluids and withholding nephrotoxic drugs. His alanine transaminase (ALT) was raised at 96 U/L (10–60), with normal bilirubin.

‘Pyrexia of unknown origin’ testing was commenced, with the history of exposure in Central America alongside other causes of renal failure focusing investigations on travel infections including possible leptospirosis. Hepatitis A/B/C/E, leptospirosis, fungal polymerase chain reaction (PCR), Cryptococcus, respiratory viral throat swab, rickettsia, Q fever, parvovirus, Epstein-Barr virus (EBV), mycoplasma, arbovirus, strongyloides and toxoplasma were all negative. Cytomegalovirus (CMV) was PCR negative. Computerised Tomography (CT) chest, abdomen and pelvis and a Magnetic Resonance Imaging (MRI) head were also performed, both normal. Lumbar punctures were attempted but unsuccessful and subsequently refused. Cefuroxime (7-day course) was commenced for broad bacterial cover including central nervous system infections.

A test for syphilis was requested, which came back as positive for total antibodies, Immunoglobulin M, treponema pallidum particle agglutination (TPPA) and rapid plasma reagent (RPR: Titre > 1:128). Full syphilis treatment was commenced, with an injection of long-acting benzylpenicillin as per British Association for Sexual Health and HIV (BASHH) guidelines for primary/secondary syphilis management [5] as well as supportive therapy.

After 9 days, symptoms resolved and there was further renal recovery. In follow-up discussion with the patient and their partner, they now reported sexual contact with an unknown case of syphilis, from a UK host. Following treatment, retroviral therapy was re-commenced.

## Discussion and conclusion

Given the history, in this case differential diagnoses could be a travel related condition such as malaria, viral (dengue), as well as leptospirosis although none fit exactly. Other differentials would include an acute hepatitis. The unusual and concerning feature is the acute renal failure, hence the initial importance of excluding and treating leptospirosis.

Initially the disease picture fitted well with leptospirosis – an incubation period of 6–29 days fits with the symptom emergence in this case [6]. Headaches and myalgia also occur in this disease [6]. Despite this gentleman having painful eyes, leptospirosis characteristically causes conjunctivitis (without exudate) which was not observed in this case [6]. The classic severe leptospirosis picture (known as Weil’s disease) with grossly deranged liver function tests, jaundice and renal failure [6] was not exhibited fully. Furthermore, the first phase of leptospirosis lasts 3–7 days [7]; this gentleman’s symptoms emerged and remained over a longer period.

A pre-renal cause of Acute Kidney Injury (for example vomiting leading to hypovolaemia) was considered, however full resolution only occurred following administration of benzylpenicillin and not solely through administration of intravenous fluids and holding nephrotoxic drugs, suggesting syphilis directly impacted the AKI.

First presentation of secondary syphilis would fit this disease timeline as it typically presents 4–10 weeks following the initial infection. Those co-infected with HIV and syphilis are thought to be more at risk of complications from syphilis such as hepatitis, meningitis and renal disease [2].

Glomerulonephritis and nephrotic syndrome (both renal causes of AKI) are cited as rare causes of early (primary/secondary) syphilis by NICE [2]. Literature searches revealed less than 20 reported cases of renal syphilis between 1992 and 2012, the majority of which appear in latent syphilis [8]. Presentation of renal syphilis includes mild, transient albuminemia [9] and glomerulonephritis leading to proteinuria, something not seen in this gentleman [8].

One case (2014) of AKI describes a 46-year old man with nephrotic syndrome and hepatitis in early syphilis [10]. Another describes syphilitic nephropathy, both with significant proteinuria, in two patients with HIV co-infection [11]. Membranous nephropathy was demonstrated in kidney biopsies in both cases. One case in 1991 describes nephrotic syndrome in a gentleman presenting with genital symptoms and found to have HIV and syphilis co-infection [12]. Rarer presentations of syphilis are more likely to occur as the condition becomes more common.

Previous reports conclude that syphilis nephropathy is immune complex driven through deposition of anti-treponemal antibodies and antigens [13]. AKI in the context of primary/secondary syphilis is reversible, as opposed to tertiary syphilis where renal damage is often permanent.

With hindsight, including syphilis in the first set of screening tests may have led to an earlier recognition of syphilis, although the sexual history and presentation were very atypical.

This case shows that primary/secondary syphilis is a reversible cause of AKI and adds to the knowledge base of a few cases with similar presentations. A delay in diagnosis occurred due to an initial focus on tropical diseases. Whilst this is reasonable given the history, a lesson to be learnt from this case is the importance of taking a sexual history and testing (with consent) in patients with infective symptoms – early diagnosis of syphilis and prompt management can quickly resolve complications such as AKI. This is especially pertinent in at-risk groups, regardless of the history, given the high rise in syphilis rates in recent years. Prompt diagnosis and management alongside physicians’ awareness of syphilis as a differential and its potential complications can improve patient outcomes.

## Data Availability

Not applicable.

## Abbreviations

MSM: Men who have sex with men
HIV: Human Immunodeficiency Virus
AKI: Acute Kidney Injury
NICE: National Institute of Clinical Excellence
ARV: Anti-retro-viral medicine
EBV: Epstein-Barr Virus
CMV: Cytomegalovirus
TPPA: Treponema pallidum particle agglutination
BASHH: British Association for Sexual Health and HIV
